# Evaluation of Gingival Phenotype in the Early Transitional Dentition Phase in Children—Comparison of Three Non-Invasive Methods

**DOI:** 10.3390/jcm12185897

**Published:** 2023-09-11

**Authors:** Agnieszka Kus-Bartoszek, Mariusz Lipski, Anna Jarząbek, Joanna Manowiec, Ewa Marek, Agnieszka Droździk

**Affiliations:** 1 Laboratory of Paediatric Dentistry, Pomeranian Medical University in Szczecin, Powstancow Wlkp 72, 70-111 Szczecin, Poland; kusia33@poczta.onet (A.K.-B.); anna.jarzabek@pum.edu.pl (A.J.); joanna.manowiec@pum.edu.pl (J.M.); 2 Department of Preclinical Conservative Dentistry and Preclinical Endodontics, Pomeranian Medical University in Szczecin, Powstancow Wlkp 72, 70-111 Szczecin, Poland; mariusz.lipski@pum.ed.pl (M.L.); ewa.marek@pum.edu.pl (E.M.); 3 Department of Interdisciplinary Dentistry, Pomeranian Medical University in Szczecin, Powstancow Wlkp 72, 70-111 Szczecin, Poland

**Keywords:** gingival thickness, children, ultrasonic measurements, visual method, TRAN method

## Abstract

Gingival phenotype (GP) is determined based on the thickness and width of the gingival tissue. An evaluation of GP is essential for adequate treatment planning and outcome monitoring, including orthodontic treatments in a paediatric population. The present study aimed to compare the reliability of the visual and TRAN methods with that of the ultrasound biometer measurements in the early transitional dentition phase. One hundred ninety three generally healthy, 7-year-old children were examined. An assessment of GP was performed by a paedodontist and a periodontist. The average thickness of the gingiva was 0.76 ± 0.36 mm, which was classified as a thin GP. The agreement between a visual assessment and the biometric ultrasound measurements reached the highest (94%) level when assessing a very thin GP (Spearman’s rank correlation coefficient r = 0.37, *p* < 0.01). Similarly, 99% agreement in the diagnosis of a thin GP was recorded for the TRAN and ultrasound methods (Spearman’s rank correlation coefficient r = 0.49, *p* < 0.001). In total, 86% of cases diagnosed as having a thick GP using the TRAN method turned out to be thin according to the ultrasound measurements. The dentist’s specialization and professional experience in the assessment of GP were irrelevant (Spearman’s rank correlation coefficient r = 0.49, *p* < 0.001). All methods tested in the present study were proven to be easy to perform and well accepted by the children. The visual assessment and TRAN methods, despite the fact that they enabled the diagnosis of a thin GP (crucial for treatment planning), cannot be recommended during the teeth replacement period. A misdiagnosis of thick GP may deprive a young at-risk patient of special supervision, which may develop into mucogingival deformities. A biometric ultrasound, although expensive, allows for reliable assessment of the gingiva thickness when needed.

## 1. Introduction

Gingival thickness (GT) and keratinized tissue width (KTW) are two important parameters used to categorize gingival phenotype (GP). The American Academy of Periodontology Best Evidence Review by Kim at al. indicates that both components vary within and between individuals and are positively correlated [1]. Reportedly, the most common cutoff dimension to separate thick and thin GP is 1.0 mm [2,3,4,5]. There is current evidence that subjects with thin and narrow gingiva tend to have a higher tendency to bleed than individuals with a thick gingival phenotype and demonstrate more recessions of higher severity [1]. Gingival phenotype can be modified by environmental factors and clinical interventions such as orthodontics or autogenous gingival grafting procedures [2].

An assessment of periodontal soft and hard tissues during the period of teeth replacement in children can be of importance for paedodontists; periodontists; and in particular, orthodontists. An orthodontic treatment plan should include a careful evaluation of the mucogingival complex in order to identify children at risk of developing mucogingival deformities due to their thin biotypes [6]. The early period of mixed dentition, in which some malocclusions are revealed, is an excellent opportunity to introduce interceptive orthodontics. This therapy can eliminate or reduce the severity of a developing malocclusion [7]. Despite the fact that, in the active phase of an orthodontic treatment, certain structural changes take place, well-planned and properly conducted orthodontic treatments do not have any constant harmful effects on the periodontal tissues [3]. Exceptional attention is required in patients with the thin gingival biotype, in whom labial tooth displacement may lead to bone dehiscence or fenestration and consequently to a recession appearance [8,9]. The thickness of the gingiva can play a crucial role in this process [10,11].

In the past decade, the gingival biotype/phenotype has been analysed in numerous studies; most of them included the upper anterior teeth in adult subjects. However, there are limited data regarding the lower incisors in children [12,13,14]. In total, 39% of the children in the studied population (children up to 16 years of age) demonstrated thin gingivae according to the probe visibility approach, 15.3% and 49.3% in primary and permanent dentition, respectively [12]. According to the ultrasound biometer measurements, 95.2% of seven-year-old children and 93.9% of nine-year-old children have thin gingivae [14]. Distinct results were obtained in three age groups of an Asian population: 4–6 years old (deciduous dentition), 7–13 years old (mixed dentition), and 16–25 years old (permanent dentition) using invasive measurements, with 1.06 mm, 1.30 mm, and 1.08 mm, respectively [13].

Despite the variety of methods for assessing the thickness of the gingiva, only some of them are suitable for children. Invasive measurements carried out with an injection needle, periodontal probe, or endodontic instruments with a stopper require prior anaesthesia, and therefore, they are not recommended for young patients [15,16,17,18,19,20]. ST-CBCT (soft tissue cone beam computed tomography), although providing information on the thickness of the gingiva and the thickness of the labial bone plate, is not recommended for routine diagnosis in children [21,22,23]. Non-invasive methods like visual assessments or TRAN could be considered in paediatric patients. However, visual GP evaluations based on GP characteristics, thin (thin gingiva, slender teeth, narrow zone of keratinized tissue, and scalloped gingival margin) and thick (thick gingiva, quadratic teeth, broad zone of keratinized tissue, and flat gingival margin), have not been used in children so far [5,24]. The TRAN method, involving the observation of periodontal probe visibility through the tissues while inserted into the gingival groove [4,25,26,27,28], was tested only in French children under 16 years of age [12]. Biometric ultrasound measurements are a promising method for children but seems to have been tested only by our research group [29]. 

The aim of the study was to compare the effectiveness and reliability of the visual and TRAN methods in relation to those of the ultrasound biometer measurements in children in the early transitional dentition phase. 

## 2. Materials and Methods

### 2.1. Study Design, Ethical Approval, and Setting

This study was approved by the local ethics committee (KB-0012/72/17) and was performed in accordance with the Declaration of Helsinki. All study participants provided oral consent, preceded by written informed consent from their parents. This cross-sectional study was conducted in four randomly selected primary schools in Szczecin, Western Pomerania, Poland, between November 2017 and May 2018.

### 2.2. Recruitment

One hundred ninety three children at the age of 7 (101 boys and 92 girls) in the early transition dentition period were enrolled into the study. The children were healthy and free from medications that might affect gingival tissues. Their GPs were assessed in the lower arch, around the incisors, fully or partially erupted (not less than 50%). In total, 661 lower incisors were evaluated—366 central and 295 lateral; 509 teeth were permanent, and 436 were partially erupted. The other inclusion criteria were no clinical attachment loss, and healthy gingiva or at most mild gingivitis (Gingival Index (GI) ≤ 1).

### 2.3. Data Collection

GP was assessed:

I. Visually, based on the typical characteristics of the thin and thick phenotypes. An additional category was set up for clinically observed very thin gingiva, i.e., extremely delicate, translucent with clearly marked roots of the teeth. The assessment was carried out by the paedodontist during the course of specialization (AK-B) and periodontics with long-term work experience (AD);
II. Using the TRAN method, in which the University of Michigan “0” probe (Hu-Friedy, Chicago, IL, USA) with Williams calibrations 1,2,3,5,7,8,9,10 was used. The probe was inserted into the gingival sulcus labially, and when the outline of the probe was visible through the tissue, the gingiva was assessed as thin. A lack of translucency indicated a thick gingiva;
III. Using an ultrasound biometer (Pirop, Echo-Son, Puławy, Poland). The device frequency was 20 MHz, the accuracy was up to 0.01 mm, and the range of measurements was 0.25–6 mm. The measurements at each point, i.e., mid-buccal aspect of the tooth, on a long axis, halfway between the muco-gingival junction and the free gingival groove, was taken twice with a small (diameter 1.7 mm) head positioning at an angle of 90° to the bone plate. Each measurement value was the arithmetic mean of 10 correctly taken measurements calculated automatically using the device. The measurements taken with the Pirop were classified as follows:
  ≤0.69 mm—very thin GP;
  0.7–1 mm—thin GP;
  >1 mm—thick GP.

The analysis considered the compatibility of the visual assessment and the outcome of the TRAN method performed by a paedodontist using the biometric ultrasound measurements. Additionally, the visual assessment of GP performed by the paedodontist was compared with the evaluation performed by the periodontist. 

### 2.4. Statistical Analysis 

The comparison of qualitative variables was performed using the chi-square test. The analysis of changes in the two repeated measurements of qualitative variables was performed using the McNemar test (for binary categorical variables) or the Bhapkar test (for multiple categorical variables). The level of significance was set at 0.05. All data analyses were conducted using R Core Team, version 4.0.2, Vienna, Austria (2020). 

## 3. Results

All non-invasive methods of assessing gingival biotype were used in all examined subjects. None of the children refused the examination, and no one reported unpleasant sensations. 

The average thickness of the gingiva in the examined group of children did not exceed 1 mm (mean ± SD 0.76 ± 0.36, Me = 0.7, min 0.29, max 3.18), classifying the GP as thin. Fully erupted permanent incisors, both central and lateral, presented the thinnest gingivae, with average thicknesses of 0.59 mm and 0.77 mm, respectively. Teeth positioned labially in the dental arch presented thinner gingivae than those erupted in the correct or lingual position (*p* < 0.001).

### 3.1. Visual Assessment vs. Biometric Ultrasound Measurements

**Figure 1:**
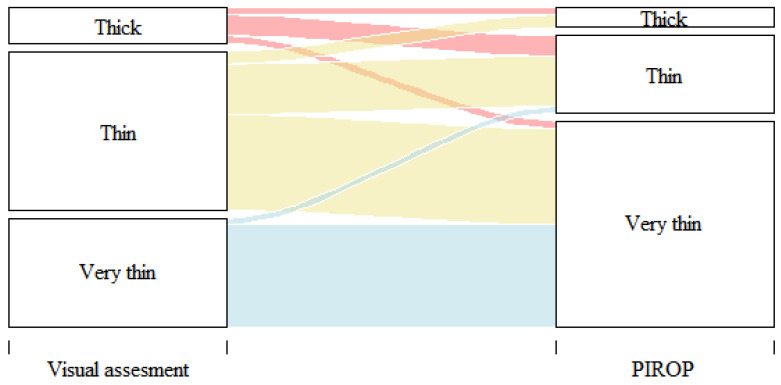
Correlation of visual assessment and PIROP measurements of GP (Spearman’s rank correlation coefficient r = 0.37, *p* < 0.01).

In the visual assessment, most cases (85%) presented a thin or very thin phenotype. The thin phenotype was diagnosed in 68 children, while the very thin phenotype was diagnosed in 97 children. The highest, 94%, agreement of the visual assessment with the biometric ultrasound measurements was shown in the case of a very thin biotype (Spearman’s rank correlation coefficient r = 0.37, *p* < 0.01) (Table 1 and Figure 1). In 17 cases, their GP was assessed visually as being thick but, in the ultrasound measurements, was classified as thin (12 children) and very thin (5 subjects). 

### 3.2. Probe Visibility through the Marginal Gingiva (TRAN) versus Biometric Ultrasound Measurements

**Figure 2:**
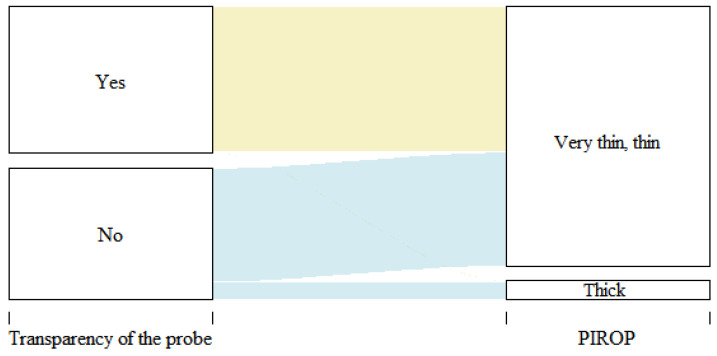
Correlation of GP assessment based on the probe visibility and the ultrasound measurements (PIROP) (Spearman’s rank correlation coefficient r = 0.49, *p* < 0.001).

During the assessment of GP with the TRAN method, in 102 cases (53%), the periodontal probe was visible through the margin of the gingiva, while in 91 children (47%), it was not visible (Table 2). The biometric ultrasound measurements in 101 out of 102 children (99.02%) confirmed thin and very thin gingivae (Spearman’s rank correlation coefficient r = 0.49, *p* < 0.001). Most cases (86.81%) in which the periodontal probe was not visible (thick GP) were classified as thin based on the ultrasound measurements. Only twelve thick GPs (13%) were confirmed using the biometric measurements (Figure 2).

### 3.3. Visual Assessment of GP around Fully and Partially Erupted Mandibular Incisors Performed by a Paedodontist and a Periodontist

An analysis of the visually assessed phenotypes around the incisors was performed separately for fully and partially erupted teeth. Complete agreement (100%) of the phenotype assessment carried out by the paedodontist and the periodontist around the fully erupted permanent incisors was noted for the thick gingiva and slightly lower for the thin (86.67%) and the very thin (72.22%) GPs. A Spearman’s correlation coefficient of r = 0.70 indicated a high correlation between the assessments (*p* < 0.001) (Table 3). The overlap in GP diagnosis with clinical confirmation around the partially erupted permanent incisors was 86.27%, 89.34%, and 74.29% for the very thin, thin, and thick phenotypes, respectively. A Spearman’s correlation coefficient of r = 0.85 indicated a high correlation (*p* < 0.001).

## 4. Discussion

During a child’s development, the jaw bones, dentition, periodontium, and the mucogingival complex undergo constant changes. The eruption of permanent teeth and the increase in height of the alveolar process contribute to the formation of correct dimensions of this complex component. Between the ages of 6 and 12, significant changes occur in the morphological structure of the periodontium, and GP also changes [19]. The transitional phase from primary to permanent dentition seems to be a critical period due to physiological thinning of the gingiva [12,29]. The thin phenotype is more prone to recession; therefore, orthodontic treatment requires very careful planning [24,30]. The maintenance of a healthy periodontium and the muco-gingival complex is determined based on the correct position of the tooth in the arch [31].

Teeth in the vestibular position have thinner and shorter gingivae and thinner alveolar bones. Those tissues are more susceptible to mechanical injury. The vestibular position of teeth in children is one of the main causes of recession onset [32]. During the developmental period, the prevalence of gingival recession is 18%, and it appears mainly around the lower permanent incisors [33]. Some of these recessions spontaneously decrease with age or even fade out as a result of tooth position improvement or as a result of orthodontic treatment [32,34]. Therefore, an assessment of the mucogingival complex as a part of orthodontic treatment planning is mandatory [6].

The present study complements the scarce literature on gingival phenotype assessments in paediatric patients. A gingival evaluation identifying patients and teeth at risk of gingival recession should incorporate an easy-to-implement, sensitive, and reproducible method for distinguishing thin and thick gingivae.

Children, due to their immaturity, both physical and mental, are unique patients, and thus, most of the available methods for GP evaluation are not suitable [13,22,23,28,35,36]. Invasive methods, as they require anaesthesia, cannot be considered in most children’s cases [20]. CBCT, which shows high reproducibility and repeatability [23], can be used for the evaluation of the cortical bone and facial gingiva [21]. However, this method cannot be used routinely and to control the treatment results in children as enhanced exposure to radiation may adversely affect further development [37].

Other methods dedicated to children regarding an early diagnosis of thin gingiva such as whitening of the attached gingiva with coronal labial traction (GW test) and visibility of the gingival blood supply (BS test) do not allow for real measurements of gingival thickness [12].

The evaluated visual assessment method in the present study did not require any additional tools; it was easy to carry out and was well accepted by the children. Phenotype was assessed on the basis of typical characteristics of the thin and thick phenotypes. An additional category was designated for the clinically observed, almost transparent gingiva. Fibrous, dense gingiva was classified as thick, while delicate and friable gingiva was classified as thin. This method, although easy to implement, is subjective and does not provide accurate measurements.

In our study, the correlation between the visual assessment and ultrasound measurements was analysed. The greatest agreement was found in the assessment of the very thin phenotype (≤0.69 mm), which was visually detected in 68 cases, and 94.12% of these cases were confirmed using the ultrasound measurements. The agreement in the assessment of the thin and the thick phenotypes was much lower, 31.96% and 22.73%, respectively. The results indicated that a visual assessment during the period of tooth replacement may be reliable only in cases of very thin gingiva. This is in line with the conclusion of Cuny-Houchmand et al., drawn from the examination of adult subjects. The authors suggested that this easy visual inspection is not an accurate method for identification of the gingival biotype and can be considered to have low accuracy and high intra-examiner variability [24].

Kan et al., compared three methods: visual assessment, periodontal probing, and direct measurements. After an examination of the biotypes in the maxillary teeth, it was stated that the visual method was subjective and not sufficient as a predictor for proper diagnosis and treatment planning prior to dental procedures.

The current study also compared the correlation of the visual assessment performed by a paedodontist with limited experience in the profession and by an experienced periodontist. Complete agreement was reached in the evaluation of cases with thick phenotypes around the fully erupted permanent teeth. The agreement in the assessment of very thin and thin gingivae were 72.22% and 86.67%, respectively. Around the partially erupted teeth, the agreement of the assessment was 74.29%, 86.27%, and 89.34%, respectively.

In accordance with the studies by Cuny-Houchmand et al., and Eghbali et al., a clinician’s experience and dental specialty do not affect the accuracy of a visual phenotype assessment [5,24].

The TRAN method (probe visibility) was the second method tested in the present study to assess GP in children. This minimally invasive method was also very well accepted by the examined patients. The agreement between the results of this assessment and those of the ultrasound measurements for gingival thickness was analysed. The visibility of the periodontal probe inserted into the gingival sulcus indicated the transparency of the marginal gingiva, and a thin phenotype was noticed in 102 participants of the study. The ultrasound measurements confirmed the accuracy of this assessment in 99.02% cases. In 91 children from the study group, the probe was not visible, which suggested a thick phenotype. However, the ultrasound measurements confirmed this thick phenotype in only 13.19%. It can therefore be concluded that the TRAN method is not reliable in assessing the phenotype during the period of teeth replacement. The visibility of the probe indicated a thin gingiva, while the lack of visibility did not guarantee the presence of a thick gingiva. This could be explained by the changes observed during the eruption period in the marginal gingiva, which is thickened and rounded, and the discussed method of GP assessment refers to this anatomical part of the gingiva.

Kan et al., compared this method with the direct measurement approach using a slide calliper in adults and found no difference [4,38]. The authors stated that this method is reliable and objective in evaluating GP, contrary to our study involving children.

De Rouck et al., used this method to assess gingival thickness in 100 medical students. They proved that this method is highly reproducible, with 85% agreement between repeated measurements and a corresponding k of 0.70 (*p* = 0.002) [26]. Shrestha et al., chose this method to assess gingival biotype in 250 patients and found it to be easy to perform, minimally invasive, and suitable for routine use [25].

The biometric ultrasound measurements with Pirop in the present study were very well accepted by the children due to the small size of the device’s head and the short examination time. The examination did not require the administration of anaesthesia. Many authors suggested that ultrasound measurements are reliable and reproducible in the hands of a trained clinician [16,39,40,41].

Furtak used the analysis of variance method (% R&R) to quantify the percentage of repeatability and reproducibility of the ultrasound measurements, which was 8.4% [39]. According to the Measurement Systems Analysis (MAS) standards, R&R values below 10 are acceptable. Thus, the authors confirmed the usefulness of this device for gingival thickness evaluation. However, the authors emphasized the importance of experience and training. They also recommended measuring the thickness several times at a given point to improve the accuracy of the measurement.

Muller et al., in a group of 25 patients aged 21–53, compared five measurements of the thickness of the gingiva with the use of an ultrasound Pirop and the transgingival probing method. The repeatability of the measurements using biometric ultrasound was higher than that of the measurements using an invasive method (SD = 0.14; 0.13–0.17 mm vs. SD = 0.20; 0.17–0.23 mm, *p* < 0.001). The superiority of the ultrasound measurements was also demonstrated by other authors [16,17,39,40] who pointed out an overestimation of the measurements and invasiveness while using transgingival probing.

A limitation of this study is the subjective nature of both tested methods. Biometric measurements can be recommended in children, but the price of the devices and the need for training and experience can be an inconvenience.

## 5. Conclusions

All methods tested in the present study proved to be easy to perform and well accepted by the children. A substantial part of the examined paediatric population demonstrated thin gingivae.

The tested visual and TRAN methods make it possible to diagnose thin GP, crucial for treatment planning. However, the percentages of thick gingivae diagnosed according to both tests were significantly different from those of the ultrasound biometer measurements. As a misdiagnosis of thick GP at the time of tooth replacement may deprive young patients at risk of developing mucogingival deformities of special supervision, both methods can be recommended for daily practice only as the first-line diagnosis of thin gingiva. Biometric ultrasound measurements should be recommended for patients, especially those who are candidates for orthodontic treatment. A biometric ultrasound, although expensive, allows for the actual thickness of the gingiva to be measured and monitored when needed. Further searches for more convenient tests may be advantageous in identifying children and teeth at risk of gingival recession development, which is always challenging in children.

## Figures and Tables

**Table 1:** Visual assessment versus ultrasound (PIROP) measurements of GP.

		Visual Assessment		
		Very Thin *n* = 68	Thin *n* = 97	Thick *n* = 22
**PIROP**	Very thin	64 (94%)	59 (61%)	5 (23%)
	Thin	4 (6%)	31 (32%)	12 (55%)
	Thick	0 (%)	7 (7%)	5 (23%)

Bhapkara test.

**Table 2:** Assessment of GP based on the probe visibility and ultrasound (PIROP) measurements.

PIROP	Transparency of Probe	
	No *n* = 91	Yes *n* = 102
Thick	12 (13.19%)	1 (0.98%)
Very thin, thin	79 (86.81%)	101 (99.02%)

McNemara test.

**Table 3:** Visual assessment of GP around the partially and fully erupted permanent incisors performed by a paedodontist and a periodontist.

	Visual Assessment by the Paedodontist				
			Very thin	Thin	Thick
**Visual assessment by the periodontist**	Permanent fully erupted incisors	Very thin	72.22%	27.78%	0.00%
		Thin	6.67%	86.67%	6.67%
		Thick	0.00%	0.00%	100%
	Permanent partially erupted incisors	Very thin	86.27%	13.73%	0.00%
		Thin	4.92%	89.34%	5.74%
		Thick	0.00%	25.71%	74.29%

Chi-square test, very thin phenotype (≤0.69 mm), thin phenotype (0.7–1 mm), and thick (>1 mm).

## Data Availability

Not applicable.
